# Citius, Altius, Fortius vs. Slow Sport: A New Era of Sustainable Sport

**DOI:** 10.3390/ijerph15112414

**Published:** 2018-10-31

**Authors:** Ewa Malchrowicz-Mośko, Katarzyna Płoszaj, Wiesław Firek

**Affiliations:** 1 Faculty of Tourism and Recreation, Eugeniusz Piasecki University School of Physical Education in Poznań, 61-871 Poznan, Poland; 2 Faculty of Physical Education, Józef Piłsudski University of Physical Education in Warsaw, 00-968 Warsaw, Poland; katarzyna.ploszaj@awf.edu.pl (K.P.); wieslawfirek@gmail.com (W.F.)

**Keywords:** slow sport, slow movement, citius, altius, fortius, sports tourism, sustainable sport, sustainable tourism, body ecology, ecomobility, flow in sport

## Abstract

The objective of the article is to present the slow sport movement as a phenomenon developing in the postmodern era in opposition to the idea of citius, altius, fortius (Eng. faster, higher, stronger). The theoretical part of the article describes the health repercussions of slow movement and its implications for the sports industry and sports tourism. It also points to new challenges in sports management and sports tourism implemented in the slow style. The empirical part of the article aims at determining what influence the achievement of a self-set sports goal has on the degree of satisfaction with participation in a running event among runners. Could runners who did not set themselves any sports goal and ran for pleasure (according to the idea of slow sport) achieve the same degree of satisfaction as runners who set themselves an ambitious sports goal and achieved it (according to the idea of citius, altius, fortius)? The case study is the 6th Poznan Half Marathon, a cyclical, popular running event taking place in Poland. A total of 560 runners (*n* = 560) took part in the diagnostic survey conducted using the interview technique. The ANOVA Rang Kruskal-Wallis test and Dunn’s test were used in the study. The results show that athletes who did not set a sporting goal (ran for pleasure, company, atmosphere, participation, etc.) experienced the same level of satisfaction as athletes who achieved their intended sporting goal. It turns out, therefore, that sport and physical activity done for pleasure in accordance with the slow sport idea can provide the same level of satisfaction as sport practiced in the spirit of citius, altius, fortius.

## 1. Introduction

In recent decades, we have witnessed the emergence of “slow cultures” in the activities and publications of various “slow movements”—food, cities, design, and tourism may serve as examples. “Slow” represents an ethos that arose in opposition to hyperconsumption in search of alternative hedonism. The “slow movement” was born in Italy in the 1980s in response to the emerging fast food chains. Soon, the idea of slow cities such as Cittaslow and slow, sustainable tourism appeared. Slow travel is the ability to enjoy one chosen city, town, or even village. It is also slow sightseeing in one country, the use of local means of transportation, and eating in local restaurants [1,2,3]. The development of the slow philosophy trend can also be observed [4,5]. Recently, the development of the slow trend has emerged in the world of sport and is becoming an important element of slow life—the Italian lifestyle, which has spread rapidly (and paradoxically) to other countries. Also, the Swedish philosophy of life—lagom—convinces us that extreme exercise and murderous effort is not what our body prefers or what gives us the most pleasure. 

The objective of the article is to present the slow sport movement as a phenomenon developing in the postmodern era in opposition to the idea of citius, altius, fortius (Eng. faster, higher, stronger) (This phrase is not of Roman origin. It is a modern term. It was invented by H. Didone, and de Coubertin liked to use Latin as he believed this language carries a lot of meaning with few words. The phrase comes from the French words from which the hymn of the school École Albert le-Grand in Arcueil began. More about the origins of this phrase can be found in Section 3). The theoretical part of the article describes the health repercussions of slow movement and its implications for the sports industry and sports tourism. It also points to new challenges in sports management and sports tourism implemented in the slow style. The description of sport and tourism as important elements of social life was made in the article from the perspective of representatives of the Western cultural circle. For people from other cultural backgrounds, especially from low-income countries, the perspective of the phenomena from the borderline of leisure time described in the article may be different. It is also worth adding that in some cultures the phenomenon of travel has not developed at all and has not been recognized as a social value. Similarly, in the world of sport—for example, in some countries of Islamic culture—sports activity is even stigmatized. 

The empirical part of the article aims at determining the influence that a self-set sports goal has on the degree of satisfaction with participation in a running event among runners. Could runners who did not set themselves any sports goal and ran for pleasure (according to the idea of slow sport) achieve the same degree of satisfaction as runners who set themselves an ambitious sports goal and achieved it (according to the idea of citius, altius, fortius)? The case study for this empirical part of the article is the 6th Poznan Half Marathon—one of the most important mass running events in Poland. 

## 2. Sport and Sports Tourism in Postmodern Times

Every cultural phenomenon, even the most enduring and universal, changes along with the historical changes in the world and social changes in values. The phenomenon of tourism has also gone a long way from the ancient and medieval pilgrimages of believers to the era of mass tourism. The phenomenon of travel has been subject to modifications in every historical era, but recently—as a result of increased mobility—these changes have become particularly visible. Analyzing the most important types of travel that have developed throughout history, it can be noted that in ancient times travel was mainly to holy places in search of sacrum—to visit healing thermal waters or to watch Olympic and other Panhellenic Games. The period of the Middle Ages was also characterized by a pilgrimage movement, but educational tourism associated with the development of universities also occurred [6,7,8,9,10]. The years of the Renaissance and Enlightenment were a time of intensive development of cultural tourism, which was related to the phenomenon called the “Grand Tour”—a long journey made by young English aristocrats to the European continent for cognitive and educational purposes [11,12]. Mass leisure tourism (according to the 3S model: sea, sun, and sand) developed on a large scale in the second half of the 20th century, supporting the economic development of southern Europe [13]. The 20th century has already been described as a breakthrough, and the dynamic development of tourism is considered to be a phenomenon of that time. Tourism is becoming a mass phenomenon and an important field of economy. The 21st century is, in turn, the era of “post-tourism”, which was born as a result of social changes in values that took place in the middle of the 20th century [14,15]. 

In Western societies, the culture of duty prevailed until around that time. Social values such as diligence, duty, and piety were valued, and forms of recreation such as practicing traditional sports (e.g., skiing in natural scenery, perhaps in the Alps) dominated in tourism. In the 1970s and 1980s, the features of hedonistic culture could already be observed. Values and attitudes such as ambition, pleasure, materialistic attitudes, and an orientation towards achievements became socially recognized. In tourism, on the other hand, activities conducive to self-fulfillment—such as extreme sports or adventure travel full of pleasure and extraordinary impressions—began to play a substantial role. Since the 1990s, we have been observing the development of the culture of individualism, which in the 21st century blends with the culture of hedonism. New values are cherished, such as self-fulfillment, individualization, the search for meaning and strong emotions, spirituality and a rich internal life, and the ability to select a lifestyle that fits one’s own preferences [16,17,18,19]. Apart from the dynamic development of sports tourism, which provides its participants with strong impressions and emotions, forms of travel such as spiritual, holistic, health, medical, spa and wellness tourism, conscious cultural tourism, or tourism with a responsible attitude are also developing.

The image of postmodern tourism has revealed the processes of the socio-cultural re-evaluation taking place in the phase of transition from a “society that lacks” to a “society of excess”. Postmodern society is a post-industrial society in which basic human needs have already been satisfied [20]. The amount and importance of leisure time has also increased. Recent decades have been characterized by continuous technical progress and economic development. Changes in the economic sphere have implied several phenomena in a society in which basic goods—food, medical care, or comfortable housing conditions—have become more readily available. What was reserved for the few in the 18th and 19th centuries became the standard for the socioeconomic middle classes in the 20th and 21st centuries. In traditional societies, human activity was mainly filled by professional work. Areas such as entertainment and pleasure functioned on the principle of festivity and uniqueness. On the other hand, goods such as culture and art are widely available today [21]. The increase in income and the purchasing power of money has been combined with a reduction in the amount of time spent on work. Technical inventions have improved and accelerated professional work as well as the functioning of households, which has contributed to an increase in leisure time.

Postmodern sporting activity has increased tourist mobility among the societies of almost all continents on an unprecedented scale, and sports tourism—especially in the form of the participation of tourists in sporting events—has gained in popularity. The *World Travel Market Report* published during a conference devoted to sports tourism in London in 2011 clearly emphasized that major sporting events sometimes attract more tourists than beautiful beaches, monuments, and unusual landscapes, and as many as 80% of cities and regions that host the largest sporting events in the world have noticed that not only athletes, but also fans are becoming typical tourists increasingly often [22]. This is one of the reasons why sports tourism (especially tourism of sporting events for professional athletes and amateurs) is now one of the fastest growing branches of the tourism industry. It is estimated that 25–30% of the world’s tourism economy is nowadays comprised of sport-related travels, and forecasts of tourism development for the coming years also indicate a further increase in travels motivated by sports [23,24]. It is generally recognized that there are three types of sports tourism: active sports tourism, sport event tourism, and heritage/nostalgia sports tourism. Sports tourism involves traveling away from one’s place of residence in order to engage in sports-related activities for recreation or competition, traveling to view popular and elite sporting events in addition to traveling to visit famous sport attractions (i.e., halls of fame, sport museums, etc.) [25,26,27,28].

The tourism economy is stimulated not only by sporting events, but also by the individual travel of tourists. During their travels, they can actively develop their sporting passions away from home, which can provide athlete-tourists with a greater intensity of experience coming from participating in sporting activities away from everyday life. In today’s world, sport plays an increasingly important social and cultural role—including a role in tourism—and sports tourism should be seen as an autonomous phenomenon of our times, reflecting current trends, needs, and lifestyles. At present, there are strong links between sports and tourism that can be described as sports touristification or tourism sportification. Sports tourism is nowadays becoming an important part of the lives of people for whom sport plays a significant role in their lives. Never before has sports tourism enjoyed such popularity; the sporting and tourism activities of society have become a mega-trend in the postmodern world. 

This state of affairs has undoubtedly been influenced by the development of the healthism ideology [29], which has postmodern individuals concentrating on their own health and its behavior and protection. Recently, this trend has been particularly visible in the dynamic development of running tourism and the participation of tourists in marathons, ultramarathons, and half marathons around the world. Above all, it is the media that tell us that we have to get tired by running or going to the gym in order to be beautiful, healthy, and to live long lives. However, it should be added that there have been cases of cardiological deaths in marathons all over the world [30,31].

Interest in sports tourism is the result of social changes in values, which are increasingly focused on experience. We now live in a “society of experience” [32]. The reason for these changes is, among other things, increases in leisure time and income. Thus, the leisure time activity of societies and the range of ways that leisure time can be spent are expanding. Changes are taking place both in terms of quality and quantity. Sport is still growing not only in the number of new sports disciplines to participate in, but also in the amount of variations that are arising out of existing sports. The same is true of tourism, which is distinguished by types; even the types that already exist are subject to further diversification [33]. In the era of post-tourism, experience is becoming an extremely important factor in choosing a tourist destination. Tourists are looking for strong experiences and emotions, even the possibility to engage in risk, challenge themselves, or overcome barriers (for example, in extreme tourism in remote areas or through participation in ultramarathons) [34,35].

Also, the result of the constantly growing number of sporting events of various ranks (for professional and amateur athletes) and different scales of difficulty—as well as the continuously arising new sports disciplines that can already be practiced in almost every corner of the globe—is the dynamic growth of sports tourism, which is slowly becoming a phenomenon of our times. Today, sports tourists want to practice sports that have only been practiced by a few people before, sports of such intensity that rarely any physical organism would be able to withstand them, and preferably sports in places that are rarely visited. This is why sports tourists are becoming increasingly interested in participating in sports events such as ultramarathons, which not only provide a strong experience but also allow for the creation of an “invisible community” of people with the same (or similar) views, style, and philosophy of life. Mass events are nowadays a postmodern form of participation in social life, making one feel like a part of a larger human community [36]. They are conducive to establishing bonds and social relations, building identity and social integration. They build a community of people with similar interests and a similar sports lifestyle. This is an important integrating function of contemporary sport because we live in a culture of individualism [37,38]. However, there is a risk that mass events may only be a postmodern ritual of collective loneliness [36]. Perhaps this is one of the reasons why the importance of slow sports is growing, as they allow for the practicing of sports in smaller social groups, enabling the establishment of longer and more authentic relationships.

## 3. Citius, Altius, Fortius vs. Slow Sport—From the Ancient Olympic Games to the Era of Post-Sport

Contemporary sport, like tourism, is a socio-cultural phenomenon deeply rooted in mass culture and functioning in reality, a result of various trends. Cultural patterns are shaped by globalization, mediatization, consumerism, and other manifestations of the postmodern era. For centuries, starting from the ancient Olympic Games, sport in accordance with the idea of citius, altius, fortius was identified mainly with professional practice. It indicated the highest level of sports competition and the process of striving as part of a championship in sports forms of using the body, a competition in accordance with socially accepted rules referring to the tradition of sport in ancient Greece. People also traveled to the Olympics for aesthetic reasons—to admire the beauty of the human body and its sporting potential. The motto citius, altius, fortius was introduced into sport by Pierre de Coubertin, the restorer of the ancient Olympic Games and their main ideologist. Thanks to the success of his Olympic project, this motto quickly became the watchword for all competitive sport. This does not mean, however, that non-Olympic sport had previously favored another motto. The Frenchman did not direct, but only verbalized the spirit of modern sport. The actual author of the three-member Latin slogan was a French preacher from the Dominican order, a teacher and keen sportsman named Henri Didon (1840–1900). As Director of the École Albert le-Grand in Arcueil near Paris, he created this phrase out of three Latin words to motivate and encourage competition at a youth competition on March 7, 1891 [39]. Then, this motto was borrowed by Didon’s close friend, Pierre de Coubertin, who was present in Arcueil. Three years later, he made it the official call of the Olympic Congress, during which it was decided to re-establish ancient agons in the modern world, and the International Olympic Games Committee was set up. The expression citius, fortius, altius (in this changed order) was also included in the first issue of the *Bulletin du Comité International des Jeux Olympiques* (1894) and in the commemorative diplomas of the Congress participants. This slogan appeared again at the 2nd Olympic Congress in Le Havre in 1897. Later, it was also placed on the first Olympic flag from 1914, designed by de Coubertin, showing five intertwined colored circles interwoven with an olive branch and a ribbon with the three Latin terms. Citius, altius, fortius became the official motto of the Olympic movement at the Antwerp Games in 1920. The de Coubertin interpretation of the motto citius, altius, fortius is clear and understandable, as he expressed it directly in many articles. In the *Philosophical Foundations of Modern Olympism* (1935)—an article written at the end of his life and which can be considered an ideological testament—he distinguished a group of addressees for this motto. Not everyone can be a recipient thereof, due to obstacles like physical predispositions and certain character traits. According to de Coubertin, “out of a hundred people dedicated to physical culture, fifty must do sport; for fifty to do sport, twenty must specialize; for twenty to specialize, five must be able to achieve astonishing feats” [40]. Only these five can become Olympic athletes and have the ability to break world records. Not everyone can enter the Olympic Altis, the once sacred grove of the Olympics. Only the best crossed the Altis gates. The Olympic stadium is the modern equivalent of the Altis. Only the sports elite can enter it. The very act of obtaining an Olympic qualification (a pass to the stadium) is a confirmation of readiness for the greatest sporting feat and a commitment to the motto citius, altius, fortius. Being an Olympic athlete—according to de Coubertin—means having the capacity for “astonishing feats”. There is no room for moderation in Olympic sport. His adepts need “freedom of excess” [40]. Only records, in his opinion, will be applauded by the crowd [39]. “Citius-Altius-Fortius—this is the motto of the International Olympic Committee and the raison d’etre of Olympism” [41]. For de Coubertin, the realization of this motto is the raison d’etre of Olympism. This means that the driving and integrating force of the Olympic movement is continuous record-breaking by Olympic athletes. He was convinced of the truth of the implications: people want to watch only the highest quality sport, so they need to see a spectacle that will amaze them. The maxim citius, altius, fortius presupposes some kind of anthropological progressiveness, faith in the constant progress of human abilities and skills. Of course, de Coubertin allowed for the situation of average results—being the effect of special circumstances (bad weather, an accident, or a mistake)—occurring during the Games. However, these were to be exceptions. In the following Games, everything had to return to its upward track [42]. H. Lenk [43] called de Coubertin a “metaphysicist of competition” because of his fixation on records. The quest for records was supposed to be the result of natural instincts that were not to be suppressed. The thought of breaking a record was a thought against nature [40]. 

Citius, altius, fortius defines a modern competitive sport whose main and overriding goal is to achieve the best possible results. In axiology, an “overriding goal” refers to the fact that in the hierarchy of values, the sports performance is placed higher than other values such as the health, honesty, subjectivity, and dignity of the athlete. Some athletes treat this call literally and indeed are able to do almost everything to be the fastest or the strongest, not taking into account costs unrelated to sport [44]. The maxim receives criticism most often from humanists. W. Daume [43] called it “a most dangerous dictum” that when treated in absolute terms will certainly have “inhuman” consequences. It leads to “record-breaking” and “medalmania”, which destroy the body and have little in common with the humanism that is simultaneously being proclaimed. Sports clubs are becoming factories of “champions” fixated on victory and records [45], and the athletes themselves become aware of their own imperfection, incompleteness, imbalance, and need for improvement [46].

The considerations carried out within the ideology of the Olympic movement can be transferred directly to professional sport in general. Firstly, because the idea of exceeding one’s own capabilities and maximizing results is not only appropriate for Olympic sport, but also for any other sport. All of these characteristics could be seen in the world outside of sport and were appropriate for the entire modern era and modernizing societies competing in all possible areas of life. The whole world of de Coubertin’s times was an arena for international races, battles, and comparisons. Sport reflected only the spirit of the out-of-stadium world. Secondly, since the Athens Games of 1896, the success of the Olympic competition was not due to the attractiveness of the humanistic message of the Olympics, but was driven by the attractiveness of the sporting spectacle itself. Stadia of the world were filled with the promises of “astonishing feats”.

Today, the sporting spectacle remains an arena of excellent struggles, and rivalry between sportspersons remains an important pillar of sport. However, as a result of the far-reaching commercialization of sport, it covers almost the entire spectrum of human activities related to physical activity, including those related to recreation or entertainment. Care for one’s health and physical fitness is becoming a mega-trend in the leisure-time market, and the postmodern recreational athlete is becoming a busy, never-quite-satisfied seeker of sensations and pleasure, similar to the postmodern tourist described by J. Urry or D. MacCannell [47,48,49]. A person’s aspiration to maximize the experience both during active sports and the passive observation of a sports competition has become a visible trend in the postmodern era. In a similar manner as in the academic literature on tourism, in which the term post-tourism was introduced to diagnose contemporary tendencies, in the world of sport some postmodern diagnosticians use the term post-sport (or postmodern sport)—e.g., B. Pronger (1998), M. Atkinson (2010), and M. Zowislo (2009) [50,51,52,53]. A postmodern person is more and more often tired of just admiring sporting events in which the best athletes participate; in practice, these events are very similar to each other. It is increasingly common for postmodern people to want to take up the hardships of the sports lifestyle themselves—for example, through preparations to participate in a marathon. In her works, M. Zowislo poses the question of whether there is a true postmodern sport and whether it would be a kind of post-sport approached by extreme feats of risk-takers on the verge of life and death or by recreational and relaxing forms of amateur sport for all. Diagnoses and descriptions of contemporary philosophers, sociologists, and anthropologists who describe and place sport in postmodern culture aim to form such conclusions [53].

In the deliberations on the role of sport in the postmodern world, the psychological theory of “seeking sensations” constructed by M. Zuckerman should be mentioned. Nowadays, it is not enough for people to practice sports and recreation activities only to enjoy their free time and to participate in activities related to the use of their skills. Instead, these activities are based on increasing loads and increasing the effectiveness of their actions. It is more attractive to participate in sports and recreation situations that provide individuals with an opportunity to verify their own achievements, to constantly challenge themselves, and to achieve a high level of stimulation during their leisure time. Such a phenomenon is referred to as sensation seeking [54]. Today, it may be one of the most important factors influencing the choice and effectiveness of various forms of sports and recreation activities. It may be believed that participants of these activities are increasingly looking for extremely high stimuli associated with self-perfection and competition, and above all with experiencing exciting and intentionally controlled risks [55]. This could explain the growing popularity of leisure activities such as marathon running. Such people are looking for forms of recreation that provide impressions and emotions connected with risk, preferring to fight with the forces of nature, their opponents, themselves, or their own weaknesses. The direction of developmental changes in contemporary sports and recreation activities is described as the transition “from recreation to excitement”. We are currently witnessing the emergence of strongly individualized and often surprising social preferences in the choice of forms of sports and recreation activities, more and more often characterized by the search for new, more exciting forms of expression [55]. 

Extreme sports, which allow people to cross the borders imposed on them by nature, are a particular manifestation of physical activity today. Contemporary sports and recreation activities more and more often take forms perceived as risky, often on the verge of health risks. People are no longer satisfied with an ordinary marathon—ultramarathons are becoming more and more popular. Today’s athlete is a challenge-seeker and risk-seeker who aims to push their physical and mental limits. 

Sports activities such as skateboarding or surfing may be treated as funsport. They are performed by lots of people without the aim to compete with others. However, sometimes funsports lovers compete with themselves or with the forces of nature. Moreover, extreme sports are sometimes not a sufficient attraction for tourists. In addition, the place where they are practiced must be “extremely” exceptional—trips to practice bungee jumping to active volcanoes in Chile or diving in waters where sharks are found (Florida) may serve as examples. 

Parallel to these extreme experiments with sport, however, were also rational phenomena based on a common sense approach to contemporary sporting experience. These include the widespread dissemination of amateur, non-elite sport (“sport for all”) in which victory is no longer important and physical effort and the joy of participating in light competition is what matters. Currently, individual sports without elements of competition—often of a regional nature (ethnosports)—are becoming more and more important. A visible trend is also an increasing orientation towards specific target groups interested in sports and tourism activities. The number of participants in sport is increasing, especially in older age groups. Among these people, sport is increasingly becoming a component of their lifestyle while also playing an integrating role in their lives. As a result, of the aging of modern societies, older people are increasingly inclined towards recreational sports. Along with the progressive segmentation of the sports market due to age groups, there is a tendency to individualize and specialize sport and tourism activities, which is accompanied by an increase in requirements for sports infrastructure and pedagogical and sports advisory services. In general, better educated, more demanding participants of sports and recreation activities will demand more individualized and specialist ways of meeting their expectations. The response to the increase in individualistic tendencies in relation to these expectations includes sport and tourism offerings in which self-reflection and border experiences become inspiring components of the experience. This is the case, for example, in offerings relating to East Asian traditional physical cultures and relaxation practices, or in various “adventure” offerings in the case of adventure and extreme tourism [56]. 

Despite the development of modern sports, traditional sports will continue to be popular because many people still appreciate traditional recreation in the open air, allowing them to achieve a “flow” state. Such recreational activities include mountain hiking, hunting, fishing, aikido, etc. Traditional sports also attract people because participation in such sports does not usually take the form of participation in a sports competition, but rather a “performance”. Moreover, what counts is not winning, but the participation itself and the joy that comes from it. There are no losers—there are only winners. A good example is the recently resurrected Nemesque Games, which everyone can participate in regardless of age, fitness level, or sports skills. In Greek Nemei it is not the result that counts, but the participation, joy, and fun in the sports community that arises from it.

Most of the population does not fall into the category of elite athletes. However, most of us try to meet high requirements for physical activity. Over the decades, numerous marketing campaigns encouraging individuals to engage in physical activity have been developed. One might say that participation in the most organized sports is stagnating. There is a need for individual and group dancing classes and fitness and outdoor adventures in pursuit of health benefits, desired bodies, social bonds, and enjoyment. 

Sport is usually associated with speed and efficiency, so how can slow sport exist? The idea of slow sport reaches people who respect their bodies and do not want to harm them. They have time to listen to what their bodies have to say. They want to practice “careful sport”. They want to be aware of every inch of their bodies during exercise. Pushing one’s physical limits is an interesting experience, but it cannot be done at any price. Slow sport is an alternative that creates free space for women, men, younger and older people, people with disabilities, and people from different cultural backgrounds.

Classes such as tai chi, qigong, and yoga are nowadays seen as healthy lifestyle trends [57,58]. With an aging population, the question of providing a variety of physical culture and sporting opportunities becomes even more important. Although companies that offer practice in the spirit of slow sport are already being established, many organizations dealing with sport, physical activity, and recreation still ignore the diverse sporting needs of postmodern people. 

For slow sports lovers, the joy and pleasure of improving one’s own body in a sustainable manner counts more than speed, strength, or victory. We are at a key moment when an alternative to the cult of sport in the spirit of citius, altius, fortius is being born. Slow sport is a new approach to practicing pleasant, sustainable amateur sport where improving one’s performance is not important. It simply provides the opportunity to enjoy physical activity, preferably in the open air, close to nature, and in good company. Slow sport also refers to exerting effort in accordance with one’s own condition, health, and experience. It allows one to experience the joy of feeling exhausted and aware of what the body can do (despite age, trauma, or disability). There is still a wide range of sporting experiences—especially those from foreign cultural circles—that allow us to discover a new poetics of the body in motion. Speed does not matter here—it is a relative concept. Philosophers of sport [59] indicate that postmodern individuals pay attention to the quality of body movement and the quality of sensations flowing from the body in the present moment, allowing them to achieve a state between satisfaction and euphoria—flow—resulting from a complete dedication to the performance of a given activity. Flow is often experienced during activities such as mountain climbing, sailing, sports games, yoga, or meditation [60].

## 4. Slow Movement and Leisure Time—New Challenges

The willingness to achieve the flow state is often a reason for interest in Far Eastern sports and physical culture practices. In the 1960s, Western European countries and the United States experienced a period of fascination with Eastern cultures. This fashion continues to this day. It is particularly visible in the popularity of practices such as yoga or spiritual travel to practice yoga [61]. Many Asian movement practices meet the criteria of slow sports (tai chi, yoga, qigong, etc.). Although yoga, for example, is not treated as a sport by many, it is undoubtedly a form of not only spiritual, but also physical, development. Yoga is nowadays an important element of the tourist product of travel agencies specializing in both cultural and sports tourism. This is not only the case in foreign destinations with a long tradition of yoga, such as India. Today, yoga is treated by many people all over the world as an attractive form of fitness and a good way to improve one’s silhouette. Yoga is a cultural, spiritual, and physical activity, which in the dynamically developing culture of physical activity plays an important recreational and relaxing role, conducive to the maintenance of the health, both physical and mental, of modern individuals. Yoga is a comprehensive, timeless, and practical Indian educational, hygienic, and therapeutic system. It is already well established in the Western cultural landscape and has found a permanent place in the structures of social life. June 21 has been declared International Yoga Day by United Nations Educational, Scientific and Cultural Organisation (UNESCO) because of its profound potential for intercultural communication. Yoga is a unique and multilevel tradition and philosophy that binds body, soul, and mind. Traveling to participate in yoga also meets the criteria of spiritual and holistic tourism. Yoga tourism has developed along with the trend of traveling “to feel better”. The interest in Far Eastern sports practices has initiated a very profitable business. This interest is also used in event marketing—for example, the global clothing company Oysho organizes “yoga marathons” around the world. 

Corporal practices such as yoga and Far Eastern martial arts have a great intercultural potential. Martial arts have been involved in the cultural dialogue between the East and West for centuries [62]. However, intercultural communication is more than just getting to know traditional practices related to the culture of physical activity. It is a challenge for sport managers and slow sports tourism managers. According to the concept of culture as an iceberg [63], when we come into contact with a foreign culture we see only its visible elements and rarely notice or understand its deeper meanings. In this concept, culture is presented as an iceberg—above the water level we see only a small part of it, which is supported by a much larger but invisible part below. This is illustrated by the fact that there are visible elements of culture—e.g., architecture, art, cuisine, music, language, and sport. However, these visible elements of culture are above all a reflection of the invisible aspects of culture, constituting powerful foundations—i.e., their cultural standards and values and basic assumptions concerning space, nature, time, etc. It is, therefore, very difficult to understand culturally alien sport and to avoid shallow imitations. Deep knowledge and even more effort are needed to get to know the elements of a culture that are “under the surface of the water”.

Today, the representatives of Western societies travel to Asia often in search of health, happiness, and beauty. However, they also travel to this region for snobbish reasons. It is worth referring to the theory of “cultural omnivorousness”. This term was first introduced to the literature on cultural consumption by R. Peterson in 1992 [64]. This phenomenon began to be noticeable at the end of the 20th century as a result of macroeconomic changes in the socioeconomic and political spheres. Omnivorous consumers have a broader cultural taste and a desire to cross the established hierarchical cultural boundaries. It is a strategy for building one’s own identity and a way of distinguishing oneself from others. When researchers in Europe became interested in the term, many domains of cultural consumption were analyzed. Culinary, musical, and sports tastes were analyzed, among others. This term is extremely important for contemporary cultural theory, and the debate on the strength and direction of the relationship between socioeconomic status and cultural taste has intensified. There are also questions as to whether cultural omnivorousness generates tolerance and cultural integration [65,66].

As far as culinary tastes are concerned, what, when, how, and with whom we eat is the result of the play of several orders: natural, religious, medical, and cultural. Food is simultaneously the basis of life, a bond-forming ritual, and an intimate expression of closeness. Food can be treated in the category of distinction. The menu expands along with higher social class status, which manifests itself in varied foods, including dishes considered rare and exotic. The higher people are in the social hierarchy and the greater their education, the more willing they are to experience new things. It is in this group that the phenomenon of culinary omnivorousness occurs most often—i.e., trying different dishes. This need to try new dishes influences the dynamic development of culinary tourism, which sometimes becomes a manifestation of snobbism, ostentatious consumption, and the act of striving for prestige. Analogies can be found in the world of sport [67]. The theory of cultural omnivorousness is connected with the dynamic development of many new forms of tourism, such as sports or cultural tourism. The trend of the 21st century is the search for more and more unusual attractions by tourists, including new and unusual sports. Traveling to Asia is also a good way to stand out from the crowd. Sometimes; however, people from certain cultural circles simply do not want to share their cultural heritage with outsiders—tourists.

Bhutan is a good example of a country that has closed its culture to the world for years and is now committed to sustainable development in tourism and ecomobility. The country is trying to resist the processes of globalization—tourists eat local dishes from local products and not meals from chain restaurants. Bhutan—a small territory between India and China that is surrounded by mountains on three sides—was completely inaccessible to tourists for centuries, and today it can be described as a slow country. The situation changed only in 1972, when Jigme Singye Wangchuck took power. The king wanted to protect the country’s cultural identity, but two years after taking the throne he decided to open the borders to visitors. The “Kingdom of the Dragon”, however, cannot be explored on its own—individual tourism does not exist here. Just like everything else in Bhutan, traveling around the country occurs in an unhurried manner. You can also get to know sports culture when you visit Bhutan. National sports include Bhutanese archery, khuru (darts), soxom (the javelin throw), and digor (a game resembling shot put, horseshoes, and petanque). However, it is difficult to say how much the Western tourist is able to take from this intercultural contact and how much of their sporting achievements the Bhutanese intend to show us. 

It should also be added that promoting a Western concept of sport oriented towards competition with oneself or with an opponent in accordance with the idea of citius, altius, fortius comes with a certain risk—it can have a negative impact on different cultures. Sport is part of the universal standardization process and can therefore contribute to the disappearance of traditional forms of physical activity [68]. Today, people from Western societies are eagerly learning the techniques of Far Eastern martial arts. They travel to Asia or want to practice them in their own cultural circle. Japanese, Chinese, or Vietnamese models of behavior transferred to a completely alien background and detached from the philosophies, religions, and customs appropriate for them cannot be completely understood by the inhabitants of other continents, and their transplantation is of a superficial nature. For people brought up far from the cultural patterns of Asia, sporting technique has become the most important, and the nature of sport derived from the martial arts tradition of the East has also become fascinating. However, the question arises as to whether a European or American is able to explore the secrets of Eastern cultures and whether they are ready to make the great effort to understand the foreign philosophical and religious concepts of martial arts. Unfortunately, yoga, for example, is treated only as an attractive form of fitness and a way to improve the figure by many people around the world. Although we have access to a lot of information about yoga today, it is very difficult to understand its cultural functions, its meaning—e.g., in a magical-symbolic or sacral context—or its importance in the process of societal communication [69]. Attention is usually focused on sport as a cultural tourist attraction and not on the influence of our culture (and the limitations resulting from it) on the quality of participation in sports tourism [70].

## 5. Aim of the Study

To strengthen the theoretical reflections contained in the article, an empirical goal was also set. This goal was to determine what influence the achievement of the sports goal set by runners has on their degree of satisfaction with their participation in a running event. Could runners who did not set themselves any sports goal and ran for pleasure (according to the idea of slow sport) achieve the same degree of satisfaction as runners who set themselves an ambitious sports goal and achieved it? The case study is the 6th Poznan Half Marathon event held cyclically in Poland—one of the most important running events in this country. In the last few years, the ideology of healthism has developed in Poland. This trend is visible in sports events in which Poles are willing to participate. The scale of this social phenomenon is certainly an extraordinary phenomenon at many levels, because several interdisciplinary factors have to be taken into account. Physical activity of Poles has increased dynamically in the last two decades. Positive changes began to be noticed after the political changes in Poland in 1989. Earlier, Poles were a community that, contrary to Western societies, showed much lower physical activity. After Poland’s accession to the European Union, Poland was at the end of the list of European countries in terms of physical activity. It is currently in the middle of the list. It is a rapid growth, and now the media and politicians are trying to consolidate this trend. Social, cultural and economic factors influenced the increase of physical activity of Poles. Currently, Poles are better educated, wealthier, have more free time. Poles have moved to cities in which sports infrastructure has developed dynamically-swimming pools, fitness clubs and bicycle paths. The Poles also moved to the offices. Earlier, they did a lot of physical or agricultural work. That is why they no longer thought about running or swimming. In addition, the way of spending free time allows people to emphasize social status. Sport and sport tourism have become a characteristic feature of the middle class in Poland. Especially the fashion for running has become very visible. In Poland, the number of organized mass sporting events and their participants has grown noticeably since 2000. Poznań is an example of a city with a population of over 500,000 and a wide range of sports events on offer. More than 500 events at various levels are organized annually in the city and in the Great Poland region, which puts Poznan among the leaders of running events nationally. The Poznan Half Marathon is one of the biggest running events in the city and in the whole country. It is characterized by a rich program of accompanying cultural and recreational events for runners and supporters.

## 6. Survey Method, Procedure, and Data Analysis

In the study, the diagnostic survey method was applied with the use of a standardized interview technique (the tool was a standardized interview questionnaire). The runners were interviewed after the race. They were asked if they had set themselves a sporting goal and achieved it—that is, if they achieved the time they set for themselves before the run to reach the finish line. We tried to make the sample selection in a way that ensured the best possible representativeness of the results obtained. The scheme of simple random sampling without replacement was used. In determining the number, evidence from the organizers on the expected number of participants of the event was used. In the calculations, the formula for sample size for a finite population was used. The assumption was made that the maximum error of estimate (*e*) at a 95% confidence level should not exceed 4%. A total of 560 half-marathon runners took part in the survey (*n* = 560). The respondents’ answers were analyzed in three groups—people who achieved their sports goal, people who did not achieve their sports goal, and people who did not set any sports goals. To compare the level of satisfaction with participation in the half marathon in the three groups (G1: runners who achieved their sports goal; G2: runners who did not achieve their sports goal; and G3: runners who did not set a goal), the ANOVA Rang Kruskal-Wallis test was applied. All of the runners were asked to determine their degree of satisfaction with their participation in the race on a 10-degree Likert scale (1—very dissatisfied, 10—very satisfied). All statistical analyses were conducted using Statistica Software 10.0 (StatSoft Inc., Cracow, Poland, 2011).

## 7. Respondents

The majority of the respondents were men (61.8%) between the ages of 19–35 (80.9%) with higher education (67.9%). Most of the participants came from urban areas—cities with over 500,000 residents (41.4%) and cities with 10–100,000 residents (23.2%)—and also from rural areas (19.6%). The detailed socio-demographic characteristics of the respondents are presented below (Table 1).

## 8. Results

To compare the level of satisfaction with participation in the half marathon in the three groups (G1: runners who achieved their sports goal; G2: runners who did not achieve their sports goal; and G3: runners who did not set a goal), the ANOVA Rang Kruskal-Wallis test was applied (Table 2).

The result of the test gives rise to the hypothesis that there are statistically significant differences between the levels of satisfaction in the three groups compared (H = 19.5; *p* = 0.0001). The procedure of multiple comparisons (Dunn’s test) revealed that these differences occur between the group that achieved the sports goal and the group that did not achieve it (*p* = 0.0001) and the group that did not set a goal and the group that did not achieve the set goal (*p* = 0.0016). However, there are no differences between those who did not set a goal and those who managed to achieve their set goal (*p* = 1). The analysis is complemented by a comparison of arithmetic averages and medians in individual groups. It can be noticed that these parameters were the same among the people who set a sports goal and those who did not set a goal (Average = 8.8 points; Median = 9), while among those who did not achieve their sports goal, the average and median was about 1 point lower. 

## 9. Discussion

Thus, the achievement of the assumed sports goal has a positive impact on levels of satisfaction. However, the conclusion that setting and achieving a goal is not a prerequisite for high levels of satisfaction with participation in the event seems to be very important. The results of our research show that athletes who did not set a sports goal (those who ran for pleasure, company, atmosphere, participation, etc.) achieved the same degree of satisfaction as the athletes who achieved their intended sports goal. It turns out, therefore, that sports done in accordance with the slow sport idea can provide the same satisfaction as sports practiced in the spirit of citius, altius, fortius. According to the researchers, athletes in moments of exhaustion may experience the flow state. The authors of the book Flow in Sport report that runners who experience the flow state during a race “do not feel pain, they feel strong, take pleasure in running” [71]. According to our research, the high level of satisfaction among the participants of the run (Table 2) indicates that slow sport lovers were also likely able to achieve the flow state. We can therefore say that in the world of sport, “what is slow can be flow”.

We will now note the limitations and strengths of our study. A strength is the number of runners investigated. We do not know, however, what the result would be if we compared people running outside of a sports event. Perhaps the unusual atmosphere of the competition and the charms of the place in which it was held also had an impact on the runners’ level of satisfaction with their participation. Sports events may provide more opportunities for more intense experiences at the sensual-vital, emotional, and social levels. The higher intensity of experiences that a runner encounters may be influenced by unusual scenery and the establishment of cultural and social interactions with other runners. Participating in sports outside usual conditions can therefore create a new quality that seems to be absent to such an extent from sports activity in traditional scenery. In the future, it is worth investigating whether the place in which physical activity is undertaken has an impact on different groups of athletes’ levels of satisfaction—for example, it could be asked whether activity in a sports hall, gym, or other artificially constructed space produces different levels of satisfaction than sports undertaken in natural scenery. It would also be of interest to know how the group of participants who set a specific running time as a sporting goal would perceive satisfaction in a future running event if they did not set a time goal, and vice versa. Finally, it would be important to examine how levels of satisfaction vary among athletes in different sports, not only in running. 

## 10. Conclusions

Sports events fulfil several important socio-cultural functions in the postmodern world. The most important one includes enabling people to build a sense of connection and integration with other sport lovers, thanks to which sporting events have become a postmodern form of participation in social life. Sporting events also satisfy the desire to experience strong emotions, a need that occupies a high place in the hierarchy of postmodern human needs.

The philosophy of practicing sports in the slow style is gaining in importance because sports implemented in accordance with the slow sport idea can provide the same satisfaction as sports practiced in the spirit of citius, altius, fortius. The results of our research are important from the point of view of public health. They indicate the directions the policy of health promotion should follow. We justify that promoting slow sport should be more sustainable and also take into account the needs of people who want to play sports but do not have the ability to excel in physical exercise in the classic sense. Moreover, it is worthwhile for contemporary sports instructors and tour operators to enable people not only to learn about other cultures through access to their “slow” sports as tourist attractions, but also to facilitate their understanding, rather than only imitating foreign cultural and sports practices. Today we observe cultural diffusion in sport not only through tourism, but also through the media. Some peoples, however, do not want to share their culture with tourists. In this case, only a certain part of the culture is prepared for the needs of the tourist world. What is behind the scenes often remains inaccessible for tourists, including sports tourists.

## Figures and Tables

**Table 1:** Socio-demographic characteristics of surveyed participants.

Socio-Demographic Characteristics of the Respondents *n* = 560 Runners	%
Sex	
Men	61.8
Women	38.2
Age	
<18	2.3
18–25	40.2
26–35	40.7
36–50	12.3
51–70	4.5
71 and above	0.0
Education level	
Primary education	1.4
Vocational education	2.3
Secondary education	28.4
Incomplete higher education	20.0
Completed higher education	47.9
Employment status	
School pupil (<18 years)	4.3
Student	31.8
Professionally active	56.8
Unemployed	3.6
Retired	3.6
Place of residence (population)	
Village	19.6
City of less than 10,000 inhabitants	10.0
City of 10,000–100,000 inhabitants	23.2
City of 100,000–500,000 inhabitants	5.7
City of more than 500,000 inhabitants	41.4

**Table 2:** The Level of Satisfaction from Participation in the 6th Poznan Half-Marathon.

Group	Average	Median	Standard Deviations	H	*p*
Goal achieved	8.8 points	9	1.2	19.5	0.0001
Goal not achieved	7.9 points	8	1.8		
Goal not set	8.8 points	9	1.4		
